# The molecular genetics of nELAVL in brain development and disease

**DOI:** 10.1038/s41431-023-01456-z

**Published:** 2023-09-12

**Authors:** Meghan R. Mulligan, Louise S. Bicknell

**Affiliations:** Department of Biochemistry, University of Otago, Dunedin, New Zealand

**Keywords:** Neurodevelopmental disorders, Genetics of the nervous system

## Abstract

Embryonic development requires tight control of gene expression levels, activity, and localisation. This control is coordinated by multiple levels of regulation on DNA, RNA and protein. RNA-binding proteins (RBPs) are recognised as key regulators of post-transcriptional gene regulation, where their binding controls splicing, polyadenylation, nuclear export, mRNA stability, translation rate and decay. In brain development, the ELAVL family of RNA binding proteins undertake essential functions across spatiotemporal windows to help regulate and specify transcriptomic programmes for cell specialisation. Despite their recognised importance in neural tissues, their molecular roles and connections to pathology are less explored. Here we provide an overview of the neuronal ELAVL family, noting commonalities and differences amongst different species, their molecular characteristics, and roles in the cell. We bring together the available molecular genetics evidence to link different ELAVL proteins to phenotypes and disease, in both the brain and beyond, including ELAVL2, which is the least studied ELAVL family member. We find that ELAVL-related pathology shares a common neurological theme, but different ELAVL proteins are more strongly connected to different phenotypes, reflecting their specialised expression across time and space.

## Introduction

Embryonic development requires tight control of gene expression levels, localisation, and timing. Levels of gene expression in cells are finely balanced and are influenced and regulated by a multitude of factors. Although transcription is largely responsible for gene expression regulation, it has become more appreciated that RNA processing at the post-transcriptional level also plays an influential role in determining the functional outcome of many RNA molecules.

RNA-binding proteins (RBPs) are recognised as key regulators of post-transcriptional gene regulation, where their binding controls splicing, polyadenylation, nuclear export, mRNA stability and translation rate and decay [1]. The binding of RBPs to target RNAs occurs in a sequence and/or structure-dependent manner and is often facilitated by *cis* consensus elements primarily localised to the 3ʹ untranslated region (3ʹUTR) of mRNA transcripts [2].

RNA molecules are first exposed to an array of RBPs in the nucleus prior to being exported through the nuclear pore to the cytoplasm [3]. Recruitment of RBPs to a target molecule triggers the formation of ribonucleoprotein (RNP) complexes, which are purposefully dynamic, allowing for continual assembly and disassembly based on the surrounding cellular environment, such as stress granule assembly [4]. Once transported to the cytoplasm, interactions between the RNA and different combinations of RBPs determine localisation, stability, and translatability of mRNA species [1]. Disruptions in the machinery required for RNP assembly can result in disease, for example in spinal muscular atrophy, caused by large deletions or missense variants in *SMN*, an essential factor in RNP assembly [5]. Distinct transcriptomic profiles are required for cellular identity, and large single-cell RNA-seq studies are exposing the rich diversity of unique transcriptomes in brain cells, both during development and changes in disease [6, 7]. Post-transcriptional processes such as alternative splicing and polyadenylation contribute to the observed diversity. Often mediated by RBPs, this post-transcriptional processing can be achieved via direct interactions with RBPs or through indirect interactions with other gene expression regulators such as non-coding RNAs [8]. Post-transcriptional mechanisms regulated by RBPs are similarly fluid to accommodate changes in the cellular proteome as required. This is particularly notable in neurons, where the proteome in subcellular compartments such as dendrites or synapses is under continual dynamic control [9]. Several RBPs have been identified to play essential roles in neural development and maintenance, where disruption impacts normal brain development leading to conditions such as intellectual disability, autism spectrum disorder (ASD) or epilepsy. For example, in Fragile-X syndrome, where individuals have intellectual disability, developmental delay, and seizures, a pathogenic repeat expansion silences translation of *FMRP*, an RNA shuttling factor [10]. Chromosomal translocations or copy number variants affecting *RBFOX1* have been associated with ASD; *RBFOX1* encodes an RBP important for stabilisation and processing of proteins important for neurotransmission [11]. The ELAVL family of RBPs are considered to have pivotal roles in neurodevelopment and will be the focus for this review.

## ELAV-like protein family—a family of RNA-binding proteins

The human family of four *ELAV-like* (*ELAVL*) genes were first identified starting with ELAVL4/HuD, which was recognised as a target antigen of paraneoplastic neurological syndrome (anti-Hu syndrome) [12]. As time progressed the ELAVL family adopted their name from the *Drosophila melanogaster* homologue, *embryonic lethal, abnormal vision* (*Elav*), although the Drosophila and mammalian paralogue ELAV families have independently evolved (Table 1) [13, 14].

**Table 1:** ELAVL family nomenclature.

Species	ELAVL1		ELAVL2		ELAVL3		ELAVL4	
*Homo sapiens*	ELAVL1 HuA^*^ HuR^*^		ELAVL2 HuB^*^ Hel-N1^*^		ELAVL3 HuC^*^		ELAVL4 HuD^*^	
*Mus musculus*	Elavl1 HuR^*^		Elavl2 HuB^*^		Elavl3 HuC^*^		Elavl4 HuD^*^	
*Danio rerio*	Elavl1a HuA^*^	Elavl1b HuG*	Elavl2 HuB^*^		Elavl3 HuC^*^		Elavl4 HuD^*^	
*Xenopus laevis*	Elavl1.L	Elavl1.S	Elavl2.L	Elavl2.S	Elavl3.L	Elavl3.S	Elavl4.L	Elavl4.S
	ElrA^*^		ElrB^*^		ElrC^*^		ElrD^*^	

Independently evolved paralogues								
*Drosophila melanogaster*		Elav		Fne		Rbp9		

*Historical nomenclature.

*ELAVL1* is ubiquitously expressed, displaying a wide range of functions [15]. Predominantly involved in stabilising mRNA targets, ELAVL1 is crucial for vital cellular processes such as cell proliferation, differentiation, and stress response [16, 17]. Conversely, *ELAVL2*, *ELAVL3*, *ELAVL4* have historically been more specifically associated with neurodevelopment with expression enriched in the central and peripheral nervous system and are therefore referred to as the neural ELAVLs (nELAVL).

**Fig. 1:**
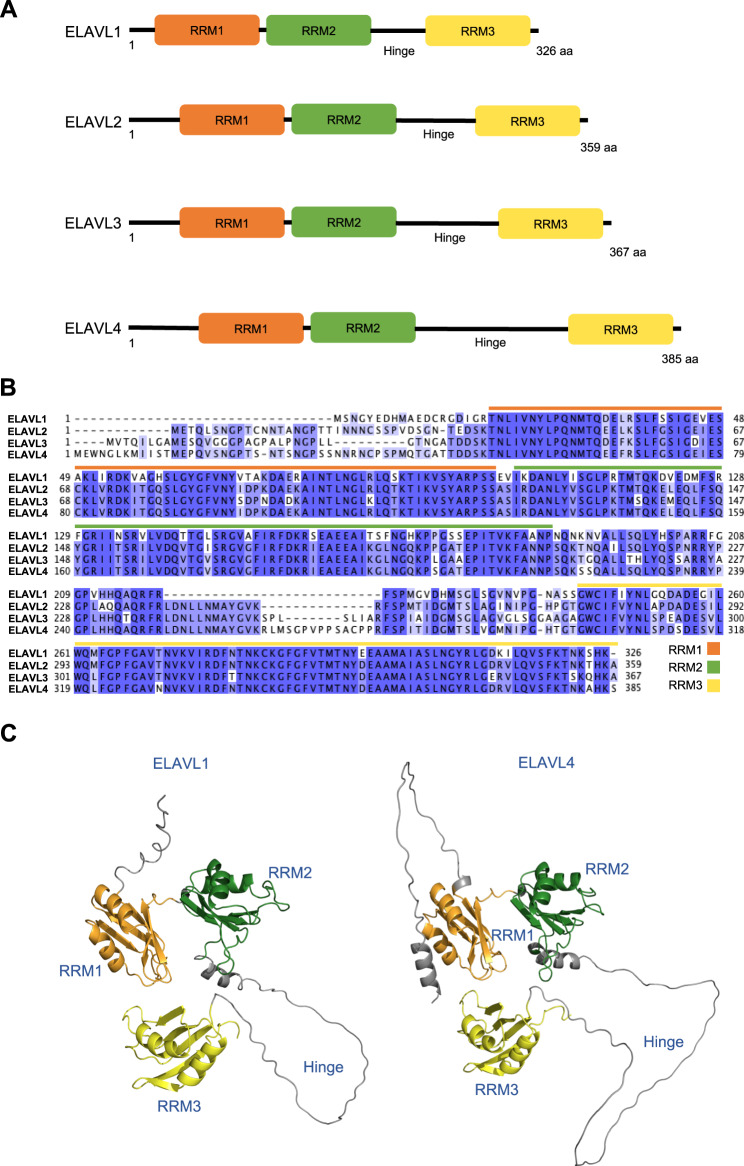
Human ELAVL protein family structure. **A** Schematic of the four human ELAVL proteins, including the three RNA recognition motifs (RRM) and hinge region. **B** Protein sequence alignment of the four human ELAVL proteins, highlighting the location of the three RRM domains. **C** AlphaFold predicted protein structures for ELAVL1 and ELAVL4. The general organisation of the protein is the same with three core RRM domains and a disordered hinge domain. Uniprot sequence IDs: ELAVL1 (Q15717), ELAVL2 (Q12926), ELAVL3 (Q14576), ELAVL4 (P26378).

RBPs typically harbour multiple (two or more) conserved RNA-binding domains that drive the RNA-protein interactions [18]. All four ELAVL proteins share a common basic structure, of three RNA recognition motif (RRM) binding domains and a hinge region (Fig. 1A). Striking sequence conservation is observed amongst the paralogues, particularly in the RRM domains (90% amino acid similarity) (Fig. 1B) [19]. Structurally, RRM1 and 2 are adjacent and located towards the N-terminus of the protein, while RRM3 is located at the C-terminal end, separated from the first two RRM domains by the hinge region (Fig. 1C). Low sequence conservation is observed at both the N-terminus and in the hinge region, facilitating the diversity observed between the different proteins [19]. Strong conservation of the RRMs suggest ELAVL proteins recognise a similar set of target RNAs, however alternative exons encoding the N-terminus alongside reduced hinge conservation likely adds to the potential to differentially interact with other cellular factors, to form a variety of RNP complexes.

Binding to RNA targets is facilitated by the RRM1 and RRM2 domains preferentially recognising AU-rich elements in the 3ʹUTR of transcripts [20]. Immunoprecipitation and RNA-sequencing studies suggest ELAVL proteins can also bind to other consensus sequences containing C-rich, U-rich and GU-rich nucleotide sequences [21, 22]. Transcripts involved in neuronal plasticity, metabolism maintenance and neurite outgrowth that contain AU-rich elements are under the regulation of the nELAVL proteins [21]. ELAVL proteins can also inhibit translation by binding to the internal ribosome entry site elements within 5’UTRs of mRNA [23].

All three of the RRM domains function to achieve the stability of RNA transcripts. RRM1 and RRM2 directly associate with the target RNA [20], while RRM3 promotes transcript stability through several mechanisms, including steric hinderance for poly(A) degrading exonucleases, association with other RNA stabilising factors and interaction with ribosomal machinery to increase translation probability [24, 25].

Nuclear export and localisation signals located in the hinge region of the ELAVL proteins are responsible for the shuttling of RNAs between the nucleus and cytoplasm [26, 27]. Shuttling of mature RNA to the cytoplasm occurs via an ELAVL-RNP complex, where the RNA is then directed towards translational machinery or is released for degradation [28].

## ELAVL proteins are active as multimers within the cell

A common mechanism observed among RBPs is the formation of multimeric protein complexes. While multimer formation can increase binding affinity of the protein to RNA targets, for some RBPs, the presence of RNA is required for efficient interaction [29]. nELAVL proteins were first observed to form multimers through Yeast-2-Hybrid experiments, with subsequent confirmation using co-immunoprecipitation of differentially tagged versions in mammalian cells [28, 30]. Both homo- and hetero-dimer formation has been observed for most ELAVL proteins, including higher order structures such as homotrimers of ELAVL4 [28]. The presence of cellular RNA molecules strongly enhanced the interaction between the ELAVL proteins, suggesting that the multimerization of the ELAVL proteins is interconnected with RNA binding. Structural, biochemical and NMR data from ELAVL1 showed that the third RRM domain facilitates the formation of homodimers and mediates protein-RNA interaction [31], with homodimerization increasing the binding affinity of ELAVL1 for its target RNAs [32].

## Elav was first identified in *Drosophila melanogaster*

The Elav family in *Drosophila melanogaster* differs from that of mammalian ELAVL and consists of three paralogous genes, *Elav*, *Rbp9* (*RNA-binding protein 9*) and *Fne* (*Found in neurons*). Elav was the first protein identified, through the characterisation of *Drosophila* mutants that were embryonic lethal and had abnormal vision [33]. Primarily localised in the nucleus, expression of *Elav* occurs throughout all stages of development and is required for the differentiation and maintenance of post-mitotic neurons [34]. The second identified member of the *Elav* gene family, *Rbp9*, which encodes a nuclear RBP expressed in neuronal cells of the adult nervous system after morphogenesis [35]. *Rbp9* is also expressed in the gonads particularly during oogenesis in the cytoplasm of cystocytes and oocytes [36]. Unlike *Elav* mutants, *Rbp9* mutants are viable, displaying no gross neurodevelopmental defects. However, mutants displayed a shorter lifespan and reduced locomotor activity, and female *Rbp9* mutants are sterile [37]. *Fne* shares similarities with *Elav* as it displays neuronal specific expression with strikingly similar transcript and protein expression patterns. Localised to the neuronal cytoplasm (as opposed to Elav in the nucleus), Fne is important for dendritic growth, cytoskeletal regulation and extracellular matrix adhesion [38]. Fne has also been linked to involved adult mushroom body development (involved in learning and memory) and courtship behaviour [39]. Null *fne* mutants are viable, but have brain defects leading to impaired behaviour such as in male courtship [39].

Understanding the function of Elav in *Drosophila* has been pivotal to unravelling the roles of human ELAVL proteins. One of the main functions of Elav is to promote the alternative splicing and polyadenylation of mRNA transcripts. The specific transcript binding and activity of each ELAV family member creates unique neuronal transcriptomic signatures. Such specificity between paralogues is also observed in other species, where studying the ELAV paralogues has helped gain knowledge into the cellular and developmental activities of these proteins [14, 40, 41].

## Expression of mouse nELAVL genes occurs in a hierarchical fashion in neurogenesis

**Fig. 2:**
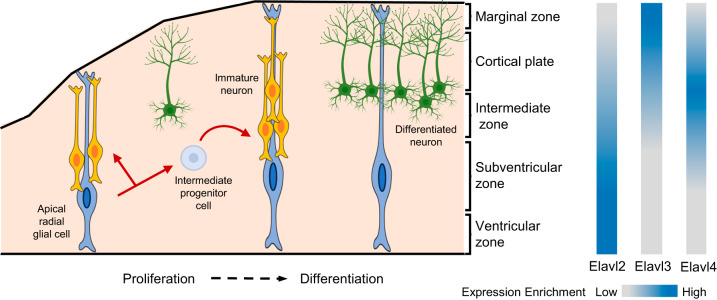
nElavl expression in the developing E16 mouse neocortex. Asymmetric division of apical radial glial cells produces an immature neuron or an intermediate progenitor cell (which in turn differentiates into a neuron), indicated by red arrows. Differentiated neurons migrate along the radial glial scaffold to the marginal zone before embedding themselves in the newly developed neocortex. nElavls displays a hierarchical expression pattern with *Elavl2* expressed early within development, followed by *Elavl4* and *Elavl3*. Dark blue indicates high expression, with light grey indicating low or no expression. Adapted from Fernández et al. 2016 and Okano and Darnell 1997 [42, 77].

Similar to *Drosophila Elav*, nELAVL are expressed in the nervous system, however they display a more specific expression pattern, with no expression in astrocytes and oligodendrocytes [42]. nELAVL proteins are primarily cytoplasmic, with a small fraction of nuclear activity, reflecting their roles (for example nuclear export, subcellular shuttling) [43]. During vertebrate neurogenesis, nELAVL genes are expressed in a specific spatial and temporal manner [42], with much insight gained from studying mouse neurogenesis (Fig. 2). In the developing mouse neocortex *Elavl2* expression is enriched in the outer layer cells within the ventricular zone and the neurons of the intermediate zone. *Elavl4* is predominately expressed in intermediate zone neurons, whereas *Elavl3* is abundantly expressed in the cortical plate, but absent from the intermediate neurons and ventricular zone cells (Fig. 2) [42]. This same spatial expression hierarchy is observed when analysing the neurons of the developing cerebellum in P9 mice [42]. Such an intricate hierarchical expression pattern suggests differing functional roles during neurodevelopment.

## Perturbation of nELAVL disrupts neurodevelopment

**Fig. 3:**
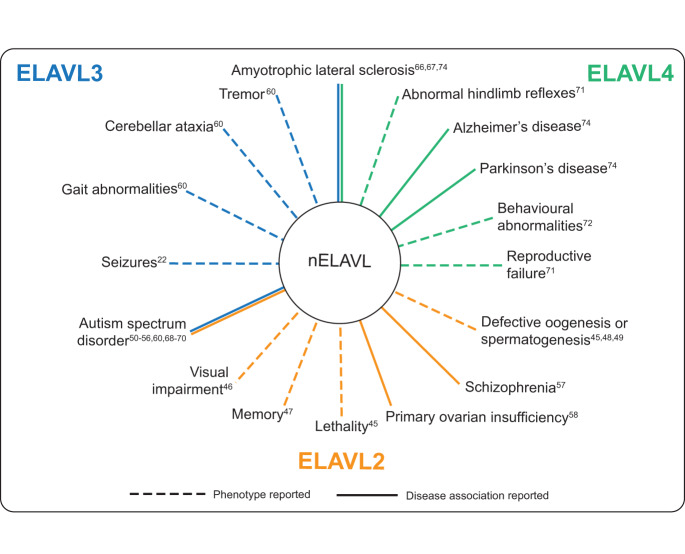
Phenotypes and diseases associated with perturbation of the nELAVL genes in animal or human. Colours indicate which of the nELAVL genes the phenotype or disease has been associated with ELAVL2 (orange), ELAVL3 (blue), ELAVL4 (green). Superscript numbers correspond to supporting literature.

ELAVL3 and ELAVL4 have been the most extensively studied amongst the nELAVL members. Knockout mouse models have expanded our knowledge surrounding the phenotypes and diseases associated with the perturbation of the nELAVL proteins and have assisted in identifying the mechanisms underpinning the different functions of the proteins (Fig. 3). Relatively less is known about ELAVL2. ELAVL2 is similar to the other ELAVL proteins in terms of structure, with no major differences within the main domains (Fig. 1A). Studies have emerged supporting both neurological and non-neurological roles for ELAVL2, which are summarised below.

## ELAVL2 plays a role in neurodevelopment

There has been limited investigation into the precise role of ELAVL2 in brain development. The International Mouse Phenotype Consortium has produced a knockout line in this gene. Heterozygous animals show no significant phenotypes in the traits analysed, but no information on homozygous knockouts was available [44]. A knockout line has been reported in the literature, and while the different genotypes were born at expected Mendelian ratios, there was an 80% lethality rate in *Elavl2*^−/−^ animals during weaning due to growth retardation [45]. No further information was available on brain development in this model.

In the developing mouse eye, *Elavl2* is expressed in multiple cell types, including retinal progenitor cells, retinal ganglion cells, amacrine cells and horizontal cells [46]. Using the Cre-loxP system, *Elavl2* was conditionally inactivated in retinal progenitor cells. RNA-seq of these cells confirmed alterations in transcription factors, including some direct Elavl2 binding targets. This caused a loss of specific subtypes of amacrine cells, which are interneurons that relay input from photoreceptors to ganglion cells. The absence of these subtype amacrine cells impacted on signal transduction to the brain, and a loss of visual acuity [46].

The relevance of Elavl2 to brain function has been studied through RNAi in a non-model organism, *Apis melifera* (honeybees) [47] - studied because the *A. melifera* genome only encodes a single member of the Elavl family, *Elavl2*, which is more similar to *fne*. Lack of paralogues has been compensated for somewhat by complex alternative splicing producing more than 40 protein isoforms [47]. Behavioural assays showed that Elavl2 is likely required for the formation of associative memory within the honeybee. However, further research is required to provide a more precise understanding of the role ELAVL2 plays in neurodevelopment.

## Emerging evidence for non-neurological roles of ELAVL2

Although primarily localised to the brain, Elavl2 also appears necessary for gonadal development, particularly oocytes. In 2014, Chalupnikova et al. reported the first functional analysis of an oocyte specific Elavl2 isoform [48]. Unlike the neuronal Elavl2 isoform, the oocyte-specific isoform is shorter due to an altered N-terminus and deletion of 13 amino acids in the hinge region. *Elavl2*^−/−^ knockout mice were generated to investigate the role of Elavl2 in the formation of primordial follicles [45]. Morphologically, the ovaries in knockout female mice were small, with no oocyte production evident in adults, but in males, examination of the testes showed no gross morphological differences when comparing null and wild type mice [45]. In females, absence of Elavl2 in female mice resulted in defective formation of the primordial follicles, causing lower yields of meiotically matured oocytes, leading to infertility [45]. It was discovered that Elavl2 promotes the translation of *Ddx6* in oocytes, which in turn directs the assembly of P-body like granules prior to the formation of primordial follicles. RNA immunoprecipitation and sequencing of Elavl2 binding targets in mouse ovaries confirmed a significant role for Elavl2 in RNA processing and cytoplasmic ribonucleoprotein granule formation [45].

A more recent study using both human and mouse testicular cell lines has investigated the function of ELAVL2 in spermatogenesis [49]. ELAVL2 levels were either reduced or increased through over-expression to investigate the consequences to proliferation and apoptosis. ELAVL2 was associated with cellular proliferation and inhibited apoptosis in both cell lines, with an increase in pro-proliferation markers and activating phosphorylation marks. Testicular biopsies from patients with non-obstructive azoospermia, a severe cause of male infertility, were also examined for the relevance of ELAVL2 to this pathology. Single-cell transcriptome data revealed that *ELAVL2* is enriched in spermatogonial cells but is reduced in non-obstructive azoospermia patients [49]. This study therefore provides evidence that whilst ELAVL2 is crucial for oocyte development, it also plays a role in spermatogenesis.

Investigations into ELAVL2 are still in their infancy, with many areas remaining unexplored. *ELAVL2* is clearly a fundamental gene required for organism development, particularly in the brain and gonads. The exact cellular roles of ELAVL2 and RNA targets under ELAVL2 regulation are yet to be fully elucidated across multiple systems.

## ELAVL2 and its relevance to disease

Given the established link between ELAVL proteins and brain development, ELAVL2 regulation was studied as a pathway of interest in neurodevelopmental disorders. To investigate the potential targets of ELAVL2, primary human neurons were depleted of ELAVL2 via RNAi-mediated knockdown [50]. Using differential gene expression analyses, transcriptional and splicing networks under ELAVL2 regulation were identified. Of the target RNAs identified, a portion of those had been previously identified as targets of other RBPs (FMRP and RBFOX1) with an established association with ASD [50]. Differentially expressed genes were identified to be involved in neurodevelopmental pathways such as axon guidance, synaptic function, and neuronal migration. Supporting this, the Simons Foundation Autism Research Initiative (SFARI) web resource lists *ELAVL2* as a candidate gene for ASD and intellectual disability, and several loss-of-function variants have been identified in large cohort sequencing studies [50–56].

In 2011, a genome-wide association study (GWAS) approach was used to determine schizophrenia susceptibility genes using three independent cohorts from Chinese and Japanese populations [57]. Analysis showed a nominal significant association between a SNP in *ELAVL2* and disease, but the significance was not maintained during replication studies or correcting for multiple testing. The specific SNP of interest, rs10491817, lies within the first intron of *ELAVL2*, but any functional consequence of the SNP has not yet been confirmed. Independent replication of this finding is required before a firm association between *ELAVL2* and schizophrenia can be concluded.

Recently *ELAVL2* has been identified as a candidate causative gene in primary ovarian insufficiency (POI) [58]. POI commonly causes infertility, but is also associated with severe health complications such as cardiovascular disease, neurodegeneration and osteoporosis. Amongst seventy families that underwent extensive genetic analysis, two siblings were compound heterozygous for two missense variants in *ELAVL2*. This is an intriguing link given the requirement for Elavl2 in oogenesis [45, 48], but given the large amount of genetic heterogeneity, further families are likely needed to confirm this as a causative Mendelian gene for POI [59].

ELAVL2 is the least studied of the nELAVL paralogues, and while there is clear evidence supporting a neurological role, ELAVL2 is obviously required in other tissues, particularly within the reproductive system (Fig. 3). A homozygous mouse model shows high levels of weaning-age lethality, so we encourage future studies understanding the pathological mechanisms causing this severe outcome. Heterozygous animals should also be examined, to better understand the consequences of partial reduction in Elavl2 on brain development and neurological functioning.

## Phenotypes associated with disruption of *Elavl*3

*Elavl3*^−/−^ null mice have no gross anatomical brain defects and were viable and fertile, although some strain-dependent lethality was observed [22]. However, both *Elavl3*^−/−^ and *Elavl3*^+/−^ mice displayed seizure activity upon EEG. As *Elavl3* is the only *nElavl* expressed in Purkinje neurons, a rotarod assay was performed to assess cerebellar function. In young adult mice, *Elavl3*^−/−^ mice had poorer balance and coordination compared to *Elavl3*^+/−^ controls, indicative of cerebellar dysfunction [22]. There were no comparisons made with *Elavl3*^+/+^ littermates, so whether there is a more subtle reduction in rotarod performance in *Elavl3*^+/−^ mice compared to wildtype mice is not clear. *Elavl3* expression is maintained during adulthood in the cerebellum. As *Elavl3*^−/−^ mice aged, progressive severe cerebellar ataxia was observed [60]. This ataxia caused an abnormal step cycle, tremor and over time, impaired postural reflexes. At the cellular level, Purkinje neurons had disrupted synaptic formation, swollen axons, and overall deficits in neuronal transport, highlighting the requirement for Elavl3 activity in these neurons [60].

## Molecular mechanisms of *Elavl3*

*Elavl3*^−/−^ mice models have been instrumental in gaining insight into the pathognomonic mechanisms in the cerebellum. High-throughput sequencing analysis of alternative splicing in the cerebellum of *Elavl3*^−/−^ mice highlighted the convergence of targeted transcripts on glutamate levels and neuronal excitability—linking RNA regulation to the seizure activity observed in *Elavl3*^−/−^ mice [22]. The physiological relevance of another target identified in the high-throughput screen, *AnkyrinG* (*AnkG*), has also been explored [22, 61]. AnkG aggregates in the axon initial segment, and through controlling ion-channel accumulation, is essential for the formation of neuronal polarity [62]. The human orthologue, *ANK3*, has been linked to multiple neurodevelopmental phenotypes [63]. Elavl3 regulates the embryonic-specific inclusion of vertebrate-specific exon 34 in the *AnkG* transcript. This exon is excluded from the canonical transcript and appears to regulate localisation of AnkyrinG at the axon initial segment, and through this, neuronal activity [61]. When analysing *Elavl3*^−/−^ mice at both 2 and 9 months of age, exon 34 was erroneously included in *AnkG* transcripts in neurons [61]. The canonical exclusion event was also confirmed in human using RNA-seq datasets from the human prefrontal cortex [61, 64].

*Elavl3* expression is upregulated during the differentiation of neural stem cells into inhibitory GABAergic neurons, in parallel with 3’UTR lengthening. Depletion of *Elavl3* during this differentiation process caused a shift towards using proximal polyA sites compared to control cells, with a concomitant delay in neural stem cell differentiation [65].

## ELAVL3 in disease

Transcriptomic profiles of motor neurons from sporadic amyotrophic lateral sclerosis nervous systems identified *Elavl3* as one of the most downregulated genes [66]. Neuropathological investigations identified a reduction of *ELAVL3* mRNA as well as nuclear depletion of protein, which was more commonly observed than cytoplasmic accumulation [67]. Similar patterns were also observed in genetic subtypes of ALS, such as *SOD1* or *C9orf72*, and in fact, in this study, ELAVL3 abnormalities were more common than TDP-43 abnormalities [67]. Using an SH-SY5Y culture model, ELAVL3 abnormalities appeared much earlier than TDP-43 abnormalities [67], suggesting ELAVL3 could have potential as a new biomarker for ALS or focus of therapeutic-targeted research.

Similar to *ELAVL2*, the curated SFARI web resource also lists *ELAVL3* as a candidate disease gene underlying ASD [52, 56, 60, 68–70]. Further confirmation is required, especially since some variants are missense, where the consequences are less clear [52]. Of note, all ELAVL paralogues show very high intolerance scores in the gnomAD datasets, supporting a requirement for a full complement of ELAVL proteins for normal development.

Studies focused on ELAVL3 has become particularly centred on putative roles in ALS, supported by mouse models showing ataxia at a later age. Further characterising the germline variants in *ELAVL3* could generate significant clues into the exact role of ELAVL3 in brain development and function. Alternative splicing is one process regulated by ELAVL3 but given the diverse roles nELAVLs can undertake in the cell, it will be interesting to investigate further potential functions of ELAVL3.

## Phenotypes associated with disruption of *Elavl4*

*Elavl4*^−/−^ mice were born at expected ratios, with no significant differences in survival, growth, or gross brain morphology [71]. Within two months however, *Elavl4*^−/−^ mice developed abnormal hindlimb reflexes, which is often associated with basal ganglia and cortical defects and in turn motor deficits. Motor coordination in adult mice was tested using the rotarod assay, with *Elavl4*^−/−^ mice performing significantly worse than WT littermates. *Elavl4*^+/−^ mice were not tested in these assays. The authors noted poor reproductive performance of *Elavl4*^−/−^ mice but did not provide any further detail on this [71]. Unlike *Elavl3*^−/−^ or *Elavl3*^+/−^ mice, *Elavl4*^−/−^ mice do not have spontaneous seizures, but are predisposed to auditory-induced seizure activity, compared to controls [72]. Behavioural tasks also suggest reduced anxiety and learning deficits [72].

Proliferation potential and differentiation capacity were tested using ex-vivo neurosphere production, where tissue from brain regions is separated into single cells and then cultivated. The number of spheres reflects proliferation capacity and potential. In cultures of *Elavl4*^−/−^derived neural stem cells, the proportion of cell spheres composed of neurons significantly decreased compared to glia or astrocytes [71]. In *Elavl4*^−/−^ embryos, there were fewer post-mitotic neurons, especially in the intermediate zone, whereas there were more proliferating cells in the ventricular zone. Together, this suggests that the number of cells exiting the cell cycle was decreased in *Elavl4*^−/−^ embryos. The overall brain size was maintained despite this reduced differentiation by an increase in apoptosis in the ventricular zone [71]. Similar results were also confirmed for neural stem cells in adult mice.

*Elavl4*^−/−^ mice have also been studied to explore the role of Elavl4 in neuron specification and dendritogenesis [72]. Staining throughout neocortical subregions revealed a role for Elavl4 in dendritic branching and lengthening. Lower layer neurons displayed fewer branch points and endings, with a reduction in overall dendritic length. Conversely, the upper neocortical layer remained unaffected. This effect was recapitulated in vitro studying neurons exposed to *in utero Elavl4* shRNA (E13). Following several days of culture, dendritic length was decreased in the neurons, despite no difference in overall dendrite number, supporting a role for Elavl4 in neurite outgrowth.

As *Elavl3*^−/−^ and *Elavl4*^−/−^ single knockout mice have been crucial in understanding the importance of the individual Elavl proteins, an Elavl3/4 double knockout mouse was generated to investigate the cumulative role of nElavl proteins in RNA regulation [22]. *Elavl3*^−/−^;*Elavl4*^−/−^ mice survived only several hours after birth, but were indistinguishable from WT littermates. The double knockout removed approximately 65% of nElavl protein in the cortex. This study has proved fundamental in understanding the specific motifs and targets of nElav proteins and their role in splicing and RNA processing and splicing. Both exclusion and inclusion of exons occurred as a result of nElavl depletion; sequence analysis suggested that U-rich sequences flanked nElavl dependent exons, whereas independent exons lacked this U-rich region. Glutamine amino acid biosynthesis was highlighted as a core pathway under nElavl regulation, and measurement of glutamate levels in cortical tissue of double knockout and WT mice, showed that knockout mice had a 50% reduction in glutamate levels [22].

## Molecular mechanisms of *Elavl4*

RNA-seq data obtained from the neocortex of 3 month old *Elavl4*^−/−^ mice has been used to identify transcriptome-wide changes to alternative splicing and alternative polyadenylation, two mechanisms known to be regulated by the nElavl proteins [73]. Several hundred genes were alternatively spliced as a consequence of *Elavl4* depletion, including many transcripts known to be bound by Elavl4. The largest proportion of events were exon skipping, with approximately equal numbers of exon inclusion versus exon exclusion in different transcripts. Pathway analysis revealed that the genes affected by alternative splicing were involved in processes such as cell death and survival, neurological disease, and nervous system development and function. When further subcategorised, pathways related to loss and viability of neurons and synaptic transmission of nervous system tissue were enriched. An analysis of alternative polyadenylation identified 53 genes which changed 3’UTR length, mostly switching to a shorter 3’UTR. A meta-analysis found a mutually exclusive grouping of transcripts that underwent alternative splicing versus alternate polyadenylation.

## ELAVL4 in disease

ELAVL4 has been associated with various neurological disorders such as ALS, Alzheimer’s disease, and Parkinson’s disease through either genetic or cellular mechanisms, and this evidence is nicely reviewed in Silvestri et al. 2022 [74]. The genetic studies have analysed signals associated with affection status or age-of-onset of Parkinson’s disease, with several common variants genotyped using SNP microarrays. While there have been attempts at replication of these association studies, the results have been mixed and are likely limited by the genetic power and approach used at the time of these studies. There are some promising avenues of research connecting cellular roles for ELAVL4 in these diseases, but these are complex, and likely intertwined with the prominent role RBPs play in neurodegenerative disease [75]. Further research in this space will help clarify where ELAVL4 dysfunction is more likely a cause or consequence in disease pathophysiology.

ELAVL4 has been the most well-studied family member, using both mouse and molecular models. Disruption to protein levels causes progressive neurological phenotypes, supported by in vitro studies finding effects on neural differentiation and outgrowth. For human disease, ELAVL4 has mainly be considered as a candidate risk gene for neurological later-onset disorders, which is appropriate given the phenotypes observed in the mouse model. These genetic studies deserve to be repeated with the larger cohorts and more sophisticated sequencing technology now available, to definitively test *ELAVL4* as a candidate gene.

## Conclusions

The nELAVL family are well established factors in brain development, but the specific roles for each gene are still being uncovered. Animal models have provided significant insight into the functions of the nELAVL family during neurogenesis, and for *ELAVL2*, other developmental systems also. Molecular studies in these models or in vitro human studies are revealing the binding targets and cellular consequences when nELAVL genes are disrupted. There is accumulating evidence supporting the relevance of this gene family to later onset neurodegenerative diseases, but data is emerging also implicating these genes in neurodevelopmental disorders, and for *ELAVL2*, a potential link to primary ovarian insufficiency. The continuing development of techniques to more sensitively interrogate RBP-RNA interactions, such as STAMP, which efficiently detects RBP-RNA interactions in single cells [76], will support further definition of binding targets and regulatory pathways involving nELAVL members, in development and disease.
